# Research status of gas sensing performance of MoTe_2_-based gas sensors: A mini review

**DOI:** 10.3389/fchem.2022.950974

**Published:** 2022-07-22

**Authors:** Jingyu Wang, Wen Zeng, Qu Zhou

**Affiliations:** ^1^ College of Materials Science and Engineering, Chongqing University, Chongqing, China; ^2^ College of Engineering and Technology, Southwest University, Chongqing, China

**Keywords:** MoTe_2_, gas sensing, transition metal dichalcogenide, additive doping, UV activation

## Abstract

Transition metal dichalcogenides (TMDs) have been widely explored for their excellent gas sensing properties, especially high sensitivity and stability at room temperature. MoTe_2_ exhibits good sensitivity and selectivity to some nitrogen-containing gases (i.e., NO_2_, NH_3_) and has received extensive attention in gas sensing. In addition, increasingly complex production environments place demands on high-quality gas sensors. Therefore, worldwide efforts are devoted to designing and manufacturing MoTe_2_-based gas sensors with faster response and recovery speed. This paper summarizes the research progress of MoTe_2_-based gas sensing, focuses on the practical measures to improve the response and recovery speed of MoTe_2_-based sensors, and discusses the mechanism. This provides guidance for exploring higher performance MoTe_2_ sensors.

## Introduction

Presently, the detection of toxic and harmful gases has received extensive attention in many fields, such as industrial production, environmental regulation and medical diagnosis (Balendhran et al., 2013). In recent years, gas sensors based on semiconducting metal oxides and conductive polymers have developed rapidly, showing good gas sensing properties in many applications (Wetchakun et al., 2011), (Wang et al., 2020). It is worth noting that such sensors require high operating temperatures (usually higher than 200°C), which brings a series of unavoidable problems, such as high energy consumption and processing difficulties (Zhu and Zeng, 2017). Therefore, it is urgent to solve the problem of high operating temperature faced by gas sensors.

Semiconductor two-dimensional (2D) transition metal dichalcogenides (TMDs) have large specific surface areas and excellent surface energy levels (Rani et al., 2019), where the specific surface area represents the total area per unit mass of the material. The larger the specific surface area, the larger the contact surface provided under the same mass condition, which helps to improve the sensor’s response (Kim et al., 2020). Among them, molybdenum ditelluride (MoTe_2_) is a new member in 2D TMDs, which has longer bonds, lower binding energy, and a smaller band gap (about 1.0 eV) by comparison (Song et al., 2016). Long bonds lead to lower binding energy, which reduces the resistance of gas adsorption on the material’s surface, which is beneficial to improve the response speed of the sensor. It also makes the desorption more rapid, helping to shorten the recovery time of the sensor. A narrower band gap can effectively reduce the bulk resistance of the material and facilitate the transition of conduction band electrons, thereby improving sensitivity. These excellent properties make it promising as a new gas sensing material. Some experiments have demonstrated the feasibility of MoTe_2_ in gas sensing.

For example, Feng et al. (2017) prepared a field effect transistor (FET) based on MoTe_2_. Experiments found that after the material was rapidly annealed at 300°C, the polarity of the MoTe_2_ field effect transistor changed from n-type to p-type, with an apparent Schottky barrier. The authors used a p-type MoTe_2_ FET to detect the concentration of NO_2_ and exhibited a large response (140%) to 100ppb NO_2_ under optimal conditions of zero gate bias with excellent reversibility at 25 °C.The authors attribute the improved sensitivity to the modulation of the Schottky barrier of the p-type MoTe_2_ FET, fully demonstrating the potential of 2D MoTe_2_.

Shackery et al. (2018) employed a micromechanical lift-off method to fabricate back-to-back diodes (SDs) with unique structures, mainly by connecting MoTe_2_-based Schottky diodes in series. It was found that MoTe_2_ Schottky diodes exhibited better performance than MoTe_2_ field effect transistors in terms of response speed and recovery time when exposed to the same concentration of NO_2_ and NH_3_ atmospheres, at 70ppb NO_2_, the response time is only 15 s. The author believes that the gas is physically adsorbed on the surface of the material based on the van der Waals force, and the charge transfer is not sustainable due to the weak van der Waals force. When the external environment changes, the charge transfer also changes, which explains the fast recovery speed of MoTe_2_ SD. It is also confirmed that the two-dimensional SD based on MoTe_2_ has good application prospects in gas sensing.

Although MoTe_2_ has made some progress, similar to other low-dimensional semiconductor transition metal dichalcogenides (TMDs), the completed work has found that gas sensors based on MoTe_2_ also exhibit slower recovery performance (Balendhran et al., 2013). This obviously limits the further application of such sensors. At the same time, some toxic and harmful gases are harmful to our health even at low concentrations (Lee et al., 2022), so it is imperative to solve the reversibility problem of MoTe_2_ gas sensors so that they can detect gases quickly, sensitively and selectively (Zheng et al., 2021). In recent years, technicians have explored various methods to improve the gas-sensing properties of MoTe_2_ with fast adsorption/desorption kinetics, such as surface modification of the material, application of gate bias (Feng et al., 2017), have achieved ideal gas sensing performance, but these methods also face a choice between performance and cost. Therefore, we believe it is of great significance to explore a method that can significantly ameliorate the adsorption/recovery speed of the MoTe_2_ gas sensor while ensuring the material has good sensitivity and selectivity. In this review, we focus on the two most practical and feasible methods for MoTe_2_ gas-sensing materials in the current research, namely UV-light activation and additive doping, and also introduce the related performance improvement mechanism.

### Research status of MoTe_2_-based gas sensors

#### UV light activation

As described above, MoTe_2_ has a narrow band gap, only about 1.0 eV, and this characteristic makes the photodetection range detectable by TMDs become wider, extending from the visible light to the near-infrared range. Recent experiments have found that MoTe_2_-based photodetectors also exhibit good responses over a wider spectral range (Su et al., 2019), which inspired us to consider the illumination factor to solve the MoTe_2_ recovery problem.

Ultraviolet light has gotten extensive attention in gas sensing as excitation energy. When the sensor is exposed to UV light with energy higher than the MoTe_2_ band gap (about 1.0 eV), electrons in the valence band are excited to migrate to the conduction band, forming electron-hole pairs with holes in MoTe_2_ (Zhou et al., 2018). Under the action of the electric field, electrons diffuse into MoTe_2_ while holes migrate to the surface. At this time, the adsorbed oxygen on the surface of MoTe_2_ will react with these holes (Kozawa et al., 2014), resulting in desorption. The remaining electrons in the MoTe_2_ channel reduce the depletion layer, increasing the gas sensor’s current (Kang et al., 2019). When the gas sensor comes into contact with the analyte, the resistance changes of the sensor due to the gas adsorption and desorption process will also be amplified under ultraviolet light. Thus, the high response and fast recovery of the MoTe_2_-based gas sensor are achieved. We summarize some information on the gas sensing performance of MoTe_2_-based gas sensors under UV light conditions, and the specific details are shown in Table 1. The sensor response is defined as (G—G0)/G0 × 100%, where G0 and G are the channel conductances before and 5 min after analyte exposure, respectively.

**TABLE 1:** Summary of gas sensing properties of MoTe_2_-based gas sensors activated by ultraviolet light.

Materials	Measured temp	UV light (254 nm)	*V*_*gs*_	Detect gas	Detection limit	*t*_response_	Response	*t*_recovery_	References
Mechanically exfoliated MoTe_2_	RT	UV = 2.5 mW/cm^2^	−60–60V	NO_2_	∼123ppt	5(min)	18%	120s	Wu et al. (2018b)
Mechanically exfoliated MoTe_2_	RT	UV = 3.5 mW/cm^2^	−60–60V	NO_2_	NA	5(min)	1300%	160s	Wu et al. (2018b)
Mechanically exfoliated MoTe_2_	RT	UV = 2.5 mW/cm^2^	−30–30V	NH_3_	∼3ppb	5(min)	790%	NA	Feng et al. (2017)
MoTe_2_-FET	RT	UV = 2.5 mW/cm^2^	−60–60V	Acetone	200ppb	5(min)	NA	180s	Wu et al. (2018a)

NA, not available; *V*_gs_, gate voltage.

**FIGURE 1:**
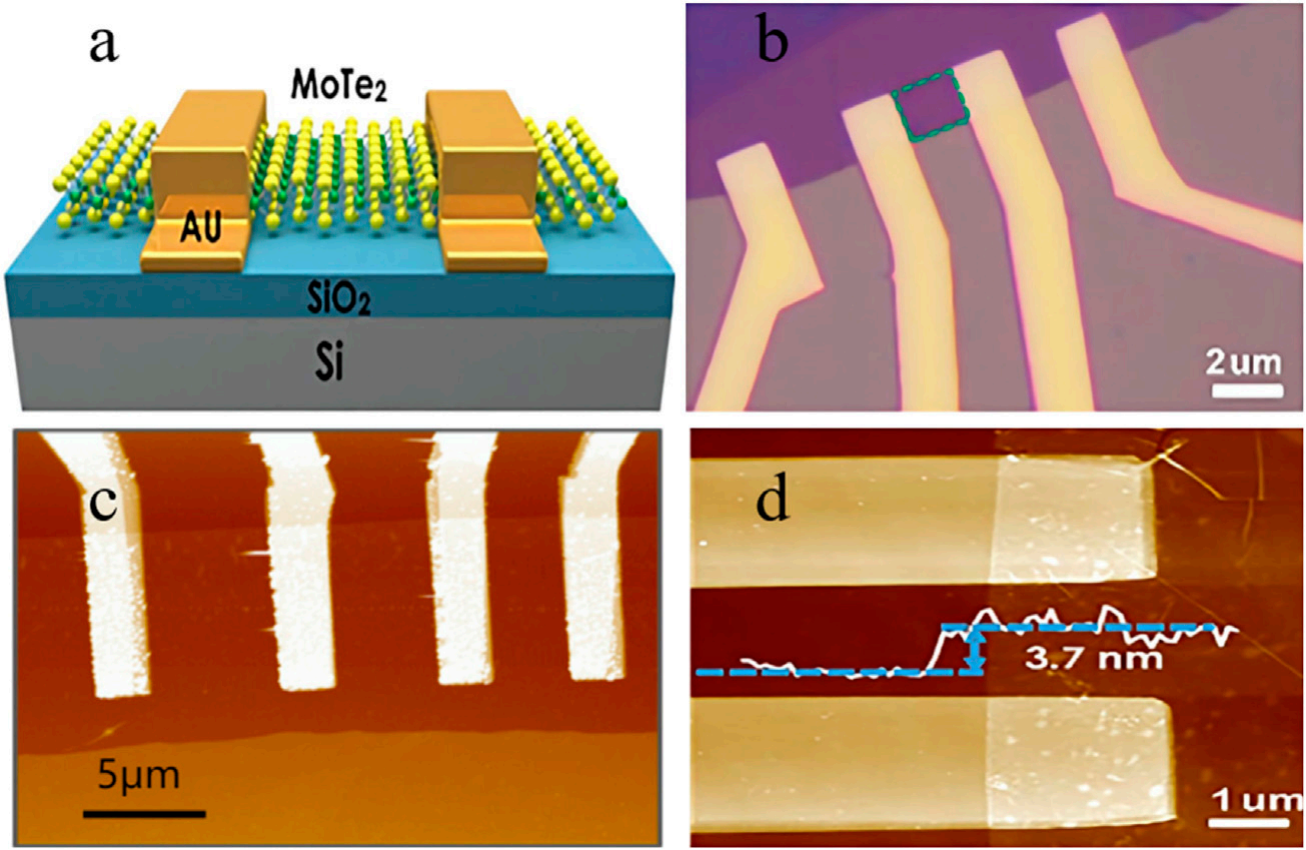
**(A)** Schematic diagram of MoTe_2_ transistor. **(B)** Optical image of the device. Scale bar is 2 μm. The sensing channel is highlighted by the green dashed rectangle. **(C)** Atomic force microscopy (AFM) topography image of the MoTe_2_ FET. Thickness of the MoTe_2_ is 3.4 nm. **(D)** AFM image of the device. The white curve is an AFM height profile.

Wu et al. (2018b) fabricated a sensor based on p-type MoTe_2_ (Figure 1B) and detected low-concentration NO_2_ under UV illumination. It was found that under the irradiation of 254 nm ultraviolet light, the adsorbed oxygen could be removed, providing more active sites for NO_2_ adsorption, which greatly improved the sensitivity of the sensor, and the detection limit reached 252ppt. What’s more, such sensors can be fully reversible in a short time under UV light, compared with even less than half of the recovery rate under the condition of no light. In addition, the MoTe_2_ sensor has excellent selectivity to NO_2_ in the atmosphere and ignores humidity interference, indicating that this type of sensor has practical application prospects.

Similarly, Feng, Z.H. and co-workers (Feng et al., 2017) reported that MoTe_2_ field effect transistors (FETs) (Figure 1A) performed control sensing detection of different concentrations of NH_3_ under both dark and 254 nm UV light irradiation conditions (Figure 1C). The experiment found that the gas sensor showed an ultra-high response speed to NH_3_ under ultraviolet light irradiation and reduced the detection limit. The results confirm that light strongly affects the gas sensing performance of MoTe_2_, and it is promising ultrasensitive sensing.

Wu et al. (2018a) fabricated MoTe_2_ FETs (Figure 1D) in another experiment to explore the effect of UV light on the detection performance of acetone. Under illumination conditions, the transistor showed the opposite response phenomenon, current increased with the increase of acetone concentration. The authors believe that acetone is adsorbed on the material’s surface to form holes, resulting in the reduction of the Fermi level of MoTe_2_ and the narrowing of the Schottky barrier width. In addition, the sensor also showed good sensitivity and a low detection limit under UV irradiation. Undoubtedly, this unique light-responsive property can discriminately detect acetone in the atmosphere and is expected to be developed into a high-performance gas sensor.

### Additive doping

In addition to UV light activation, doping additives are also a common modification measure, especially in gas sensing (Zhang et al., 2022).

Mote_2_ sheet comprises three hexagonal layers, two atomic layers of Te and one of Mo between them, and the atoms in the plane pass through normal covalently bonded (Zappa, 2017). The layers are mainly maintained by van der Waals forces, and the bonding is relatively weak.

As far as we know, doping transition metals on the surface of nanomaterials can effectively improve their electron mobility and activity (Kumar et al., 2020), especially for some noble metal dopants, which can improve the electrical properties of materials and also improve the gas adsorption properties on the surface of materials (Hou et al., 2021). Doping transition metals can effectively improve the sensitivity of gas-sensing materials, introducing more chemically active sites. In addition, selecting appropriate transition metals for doping will change the material’s bulk resistance and amplify the material’s resistance change during the gas adsorption process, thereby improving the sensor’s sensitivity (Szary et al., 2022a). The mechanism of the MoTe_2_ gas sensor is inseparable from the charge transfer between the adsorbed gas and the gas-sensing material (Agrawal et al., 2021). The gas to be tested acts as a carrier donor or acceptor during the adsorption/desorption process, thereby changing the resistance of the gas-sensing element.

**FIGURE 2:**
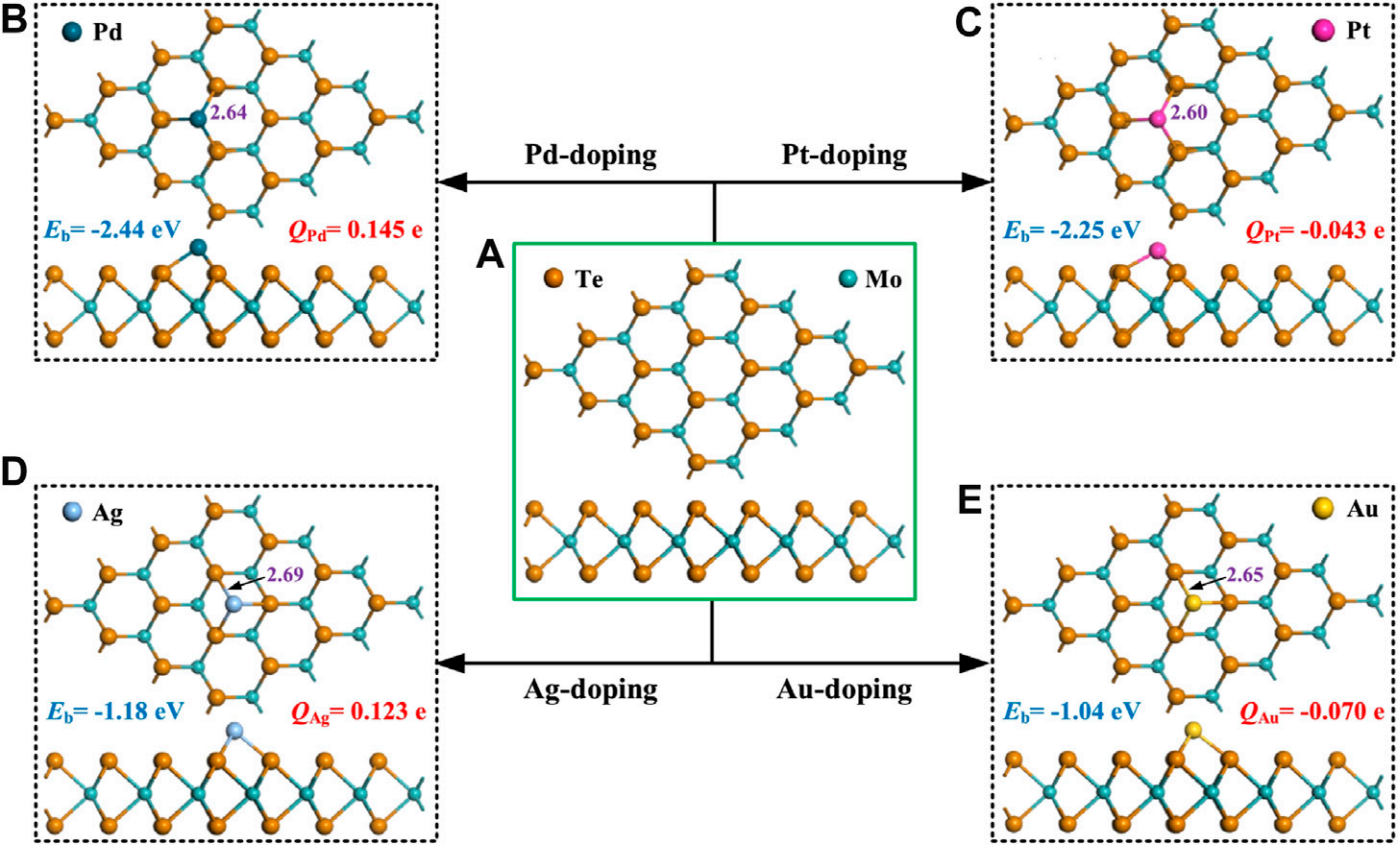
MSC of **(A)** pristine, **(B)** Pd-doped, **(C)** Pt-doped, **(D)** Ag-doped and **(E)** Au-doped MoTe_2_ monolayer. The violet values are bond lengths of TM-Te, unit in Å.

Liu, Y. and others (Liu et al., 2021) explored the effect of doping four transition metals Pd, Pt, Ag and Au (Figure 2) on the gas sensing properties of MoTe_2_ sensors based on density functional theory (DFT). When NO_2_ molecules are chemically adsorbed on the material’s surface, they show a strong electron accepting behavior, especially after Au doping. With the adsorption of NO_2_, the band gap of the Au-MoTe_2_ monolayer increases significantly, and the electrical conductivity changes greatly. It is demonstrated that the transition metal-doped MoTe_2_ monolayer has excellent potential for NO_2_ detection.

Cao et al. (2021) chose Ni with good activity and electron mobility as the doping metal, based on the first principle, to explore the gas sensitivity of monolayer Ni-MoTe_2_ to nitrogen oxide gas-sensing performance. The calculation shows that metal Ni reacts with the adsorbed gas to significantly increase the surrounding electron density, which shows a stronger binding force to NO_x_ than MoTe_2_ without Ni doped. The change of band gap during the adsorption process is more prominent, which will also lead to a change in the conductivity of the gas sensor with NO_x_ adsorption.

Panigrahi et al. (2019) used dispersion-corrected DFT to explore changes in the sensing performance of Sb-doped MoTe_2_ for nitrogen-containing gases. The substitution of Sb for Te increases the defect concentration and also enhances the binding energy. Under oxynitride conditions, substantial charge transfer occurs. At a doping concentration of 2.08%, the material exhibits a good affinity for NO and NO_2_. This theoretically proves that Sb-MoTe_2_ has broad application prospects in a new generation of gas sensors.

In other work, Szary, M.J. and others (Szary et al., 2022b) calculated the doping of Al, Si, P, S and Cl atoms on Te vacancies in MoTe_2_ based on density functional theory and discussed the sensing performance for CO and CO_2_. The calculation found that P-doped MoTe_2_ increased the charge transfer of the adsorption of the two gases, but the effect on the two gases was not correlated. The relative response value of the doped material to (CO/CO_2_) increased from 1.5 rose to 5.6, showing good selectivity for CO/CO_2_. It is expected to be further developed in the field of sensing.

In addition, Zhu, H.L. and others (Zhu et al., 2020) explored the gas-sensing properties of Rh-MoTe_2_ for SF_6_ decomposition products based on first-principles, and found that Rh-MoTe_2_ monolayer showed good adsorption performance for SOF_2_. Jiang et al. (2022) chose the metal ruthenium with high chemical activity as the doping element, and calculated the adsorption of SF_6_ decomposition products on Ru-MoTe_2_ monolayer based on density functional theory, the adsorption capacity of the system to the decomposition products was significantly improved after Ru doping. Liu, Z.C. and others (Liu et al., 2022) calculated the adsorption capacity of several harmful gases on monolayer Au-MoTe_2_ based on density functional theory, and Au-MoTe_2_ had the strongest interaction with NO_2_.

A series of ideal theoretical calculations illustrate the feasibility of additive doping in improving the sensitivity and selectivity of MoTe_2_, providing a theoretical basis for the further development of MoTe_2_-based gas sensors.

## Conclusion

In general, this paper summarizes the research status of MoTe_2_-based gas sensors and finds that applying gate bias, UV light activation, and additive doping can effectively improve the performance of MoTe_2_-based sensors. Under UV light irradiation, the oxygen adsorbed on the material’s surface is effectively removed, providing more active sites for the analyte, thereby significantly improving the sensor’s sensitivity. Many calculations based on density functional theory and first-principles have also confirmed from the theoretical level that additive doping can effectively improve the charge transfer during gas adsorption, pointing the way to the future of high-performance MoTe_2_-based gas sensors. Although the current work has achieved specific results, more efforts are needed to apply the theory to practice, and it is hoped that our work can guide for exploration of MoTe_2_-based gas sensors.
